# Drug Resistance Missense Mutations in Cancer Are Subject to Evolutionary Constraints

**DOI:** 10.1371/journal.pone.0082059

**Published:** 2013-12-20

**Authors:** Ran Friedman

**Affiliations:** 1 Department of Chemistry and Biomedical Sciences, Linnæus University, Kalmar, Sweden; 2 Linnæus University Centre for Biomaterials Chemistry, Linnæus University, Kalmar, Sweden; University of Torino, Italy

## Abstract

Several tumour types are sensitive to deactivation of just one or very few genes that are constantly active in the cancer cells, a phenomenon that is termed ‘oncogene addiction’. Drugs that target the products of those oncogenes can yield a temporary relief, and even complete remission. Unfortunately, many patients receiving oncogene-targeted therapies relapse on treatment. This often happens due to somatic mutations in the oncogene (‘resistance mutations’). ‘Compound mutations’, which in the context of cancer drug resistance are defined as two or more mutations of the drug target in the same clone may lead to enhanced resistance against the most selective inhibitors. Here, it is shown that the vast majority of the resistance mutations occurring in cancer patients treated with tyrosin kinase inhibitors aimed at three different proteins follow an evolutionary pathway. Using bioinformatic analysis tools, it is found that the drug-resistance mutations in the tyrosine kinase domains of Abl1, ALK and exons 20 and 21 of EGFR favour transformations to residues that can be identified in similar positions in evolutionary related proteins. The results demonstrate that evolutionary pressure shapes the mutational landscape in the case of drug-resistance somatic mutations. The constraints on the mutational landscape suggest that it may be possible to counter single drug-resistance point mutations. The observation of relatively many resistance mutations in Abl1, but not in the other genes, is explained by the fact that mutations in Abl1 tend to be biochemically conservative, whereas mutations in EGFR and ALK tend to be radical. Analysis of Abl1 compound mutations suggests that such mutations are more prevalent than hitherto reported and may be more difficult to counter. This supports the notion that such mutations may provide an escape route for targeted cancer drug resistance.

## Introduction

The kinase inhibitor (KI) imatinib is prescribed since 2001 to chronic myeloid leukemia (CML) patients [1]. Aimed at the tyrosine kinase domain of the abnormal chimeric protein BCR/Abl1, imatinib was the first successful targeted cancer drug. Following its remarkable success and relative safety, additional KIs are now administered for treatment of various cancers, and many others are under development [2]. The specificity of KIs varies, and some medications are used to treat several types of cancers. Imatinib, for example, is registered in Sweden not only for treatment of CML but also Philadelphia chromosome-positive acute lymphoblastic leukemia (Ph^+^-ALL), various blood syndromes, gastrointestinal stromal tumour (GIST) and dermatofibrosarcoma protuberans (DFSP). The advancement of genome sequencing techniques enables identification of patients that are more likely to benefit from targeted treatment based on the genetic profile of the tumours. Moreover, new drug targets that are distinct from kinases are being sought after. Examples include farnesyltransferase inhibitors and heat shock protein antagonists.

Unfortunately, many patients eventually become insensitive to treatment due to somatic mutations in the kinase domain of the drug targets, which prevent the drugs from inhibiting the enzymes [3], [4]. The emergence of such ‘secondary mutations’ limits the effectiveness of anti-cancer drugs in the long term [5]. The discovery that resistance mutations result in treatment failure prompted the development of second (dasatinib, nilotinib) and third (bosutinib, ponatinib) generation Abl1 inhibitors. The clinically most notorious Abl1 mutant is T315I, which is resistant to all KIs except ponatinib (recently approved in the US and EU) and rebastinib (currently studied in clinical trials). Studies with Ba/F3 cells, a convenient model system for KI development, suggest that resistance towards ponatinib and rebastinib may develop through ‘compound mutations’, i.e., two resistant mutations that occur in the same clone of tumour cells [6], [7].

It is not possible to follow the development of drug resistance mutations in single clones. This would require the ability to follow the emergence of mutations dynamically, which cannot be achieved because the samples must be sequenced, and because many of the mutations will inevitably be lost rather than fixed in the cell line. For this reason, mathematical models of drug resistance in cancer have been developed and applied to study drug resistance under different scenarios. e.g., modifying the dosage or using multiple inhibitors [8]–[12]. Such models enable the testing of various hypotheses *in silico*, often in relation to clinical findings, before progressing to cell or clinical studies.

Accounting for the evolutionary forces that lead to drug resistance is important for development of new treatment regimes that will be less likely to yield resistance. If the evolutionary landscape is constrained, small molecular drugs that target the known mutants are likely to succeed. On the other hand, if the drug target can adopt additional mutations without a significant selective pressure, any targeted treatment will eventually fail. The current paradigm in studies of resistant mutations is that those mutations occur prior to treatment. Mutation rates in protein coding genes are in the order of 10^−9^ substitutions per year per site. Even if the mutation rates in cancer cells are greater by 3–4 orders of magnitude, it is unlikely that the mutations develop during a treatment period of several years, whereas relapse often occurs within months. Thus, resistance mutations must be tolerated before treatment. To this end, two scenarios are possible. First, one may assume that all kinase domain somatic mutations are selectively neutral or slightly deleterious and therefore have a non-negligible probability to be fixed in the population [13]. This assumption may be justified by arguing that the targeted oncogene was already subject to a ‘gain of function’ mutation that lead to its primary role in the tumour, and is now relatively insensitive to further mutations. If this is true, then the only limitation on the emergence of resistance mutations is the substitution rate. In contrast, it may be assumed that the active oncogene has a biological function that may be compensated by mutations, and its evolutionary landscape is limited not only by the rate of mutation but also by purifying selection. In this case, understanding the extent of selection can lead to development of treatments that will be *a priori* less sensitive to drug resistance.

Here, I use bioinformatic analysis in order to estimate which of these scenarios is more probable, i.e., whether resistance mutations in the kinase domain are likely to be tolerated. To this end, I analysed the prevalence of such mutations in sequences that are homologous to three tyrosin kinases that are important drug targets and where drug resistance due to missense mutations presents an acute clinical problem: epidermal growth factor receptor (EGFR), anaplastic lymphoma kinase (ALK) and the kinase domain of the Abelson murine leukemia viral oncogene homolog 1 (Abl1).

### Epidermal growth factor receptor

EGFR is a cell-surface receptor tyrosin kinase (RTK) of the ErbB family. Elevated expression of EGFR is observed in cancers of various organs. Small molecule inhibitors of EGFR, such as gefitinib and erlotinib were approved for treatment of non-small-cell lung cancer (NSCLC). These molecules are competitive inhibitors of ATP binding in the active site of the receptor. The presence of several somatic mutations in EGFR, that seem to confer increased kinase activity (activating mutations, also known as driver or sensitive mutations), has been correlated with sensitivity to EGFR inhibitors [14]–[16]. Yet, some of the patients receiving tyrosin kinase inhibitors (TKI) do not respond to the treatment, and only about 5% enjoy complete remission [17]. In many cases, treatment failure is due to TKI resistance mutations, that include insertions and six different missense mutations in the tyrosine-kinase domain [17], [18]. T790M is the most common of these mutations and confers ligand independence.

### Anaplastic lymphoma kinase

ALK is an RTK that has been associated with neuroblastoma and lung cancer, through different mechanisms. In lung cancer, the fusion of ALK and echinoderm microtubule-associated protein-like 4 (EML4) leads to constitutive activation of the kinase [19]. In neuroblastoma, on the other hand, increased ALK activity is associated with ALK gene amplification, somatic and germline mutations [20]–[22]. ALK inhibitors are now being developed as drugs; the TKI crizotinib is in use in lung cancer patients carrying the EML4-ALK fusion protein. Unfortunately, secondary mutations may lead to crizotinib resistance [23].

### Abl1

Abl1 is a proto-oncogene encoding a tyrosine kinase. The fusion protein BCR-Abl leads to chronic myeloid leukemia (CML), which can be treated by TKI. 20 missense mutations in Abl have been shown to confer drug-resistance (or reduced sensitivity) to at least one of the three commercial drugs imatinib, dasatinib and nilotinib [24]. Another meta-analysis (i.e., analysis of findings from multiple experiments reported in the literature) identified 34 such mutations based on *in vitro* studies [25]. Apparently, Bcr-Abl displays a mutator phenotype, i.e., it leads to acquisition of mutations [26]. TKI Treatment apparently leads to a decrease in mutation frequency [25], indicating that mutations occur primarily prior to treatment, whereas mutant clones become dominant as the result of TKI treatment.

The results reveal that drug-resistance mutations in the tyrosine kinase domains of Abl1, ALK and exons 20 and 21 of EGFR favour transformations to residues that can be found in similar positions in evolutionary related proteins. Thus, it is demonstrated that evolutionary pressure shapes the mutational landscape in the case of drug-resistance somatic mutations. Analysis of compound mutations reveals a larger proportion of such mutations that have not been hitherto observed in related sequences.

## Results

### Epidermal growth factor receptor

Resistance to erlotinib and gefitinib has been linked to six resistance mutations [17], [18]. Analysis of sequences where the kinase domain is homologous to that of EGFR reveals that in 4 of the 6 resistance missense mutations, the same amino acid variation is observed in other sequences of related proteins (Table 1 and Table S1). These four resistance mutations are S768I, V769L, and T790M (on exon 20), and T854A (on exon21), whereas the two resistance mutations that cannot be observed as SNVs in the MSA (L747S and D761Y) are located on exon 19. This may be explained by exon 19 being a mutational hot-spot, where mutations occur in as much as 45% of the NSCLC patients [18]; it may be that the mutation rate in exon 19 is high enough that the mutations emerge during treatment.

**Table 1:** Amino acid residue variations in cancer drug resistance and drug sensitivity mutations.

**EGFR**	**Resistant mutants**	**Activating mutants**	**Total mutants**
Occurred	4	5	9
Novel	2	7	9
Total	6	12	18
**ALK**	**Resistant mutants**	**Neuroblastoma mutants**	**Total mutants**
Occurred	5	11	16
Novel	1	0	1
Total	6	11	17
**Abl1**	**Resistant mutants**		
Occurred	43		
Novel	0		

The number of residue variations that have an evolutionary origin (i.e., a similar variation that is observed in at least one homologous sequence) and those that are novel are indicated for cancer mutations in EGFR, ALK and Abl1.

On the other hand, only 5 out of 12 activating mutations are observed in the multiple sequence alignment (MSA) of EGFR and homologous proteins. This finding may be explained by considering that the activating mutations can be described as ‘gain of function’ mutations. These mutations make the kinase constitutively active, which is not desired out of context of the tumour. Hence, many of them cannot be observed as variations in related sequences.

All of the studied missense mutations are due to single nucleotide variance (SNV), and it is possible that a certain SNV is observed in the MSA because all of the possible SNVs are covered. In this case, the likelihood to identify this mutation in the MSA is 1. Indeed, All of the non-synonymous SNVs of Ser768 have been observed in the MSA. Conversely, of the six possible amino-acid replacements due to non-synonymous SNVs in position 790, only two are observed in the MSA: T790A, which is observed only in a single sequence; and T790M, which is observed in 87 sequences (31%). T790M is the most prevalent EGFR resistance mutation [18]. Thr790 is called the gatekeeper residue of EGFR, because it is located at the entrance to a hydrophobic pocket where KI bind, making it important for KI selectivity. The KI resistance due to the T790M mutation had therefore been suggested to be due to steric clashes with the bound KIs. However, it was later discovered that the T790M mutants are able to bind KIs, but remain active due to increased affinity to ATP [27]. The prevalence of Met at the same position as residue 790 in the MSA of EGFR homologues is in line with this finding. Like Thr790, residue Thr854 can be mutated to six other residues through SNVs, but only three such changes are observed: T854A (146 sequences, 52%), T854I (one sequence) and T854S (51 sequences, 18%). In this case, the mutation may indeed prevent the binding of the drug [28]. Unlike the radical mutation T854A, T854S is a conservative mutation, and would probably not lead to drug resistance. T854I is only present in one sequence. The other possible mutations T854K, T854P, and T854R may lead to drug resistance but are not found at the MSA at all, suggesting that they are selected against even if they emerge.

Further analysis of the probability to observe a given residue in the kinase domain can be obtained from the Conserved Domain Database (CDD) [29], ncbi.nlm.nih.gov/cdd. The Conserved Domain Database is a resource for the annotation of functional units in proteins. Among other data, it portrays the probability to find each of the 20 common nucleotide encoded amino acids at any position of the alignment as a log2 based position specific scoring matrix (PSSM) score. The larger the PSSM score, the more conserved is the residue at the designated position. When examining the positions of the resistance mutants in EGFR it is found that Leu747, Asp761 and Ser768 are mutated to residues that are less probable according to the conserved domain. On the other hand, Val769, Thr790 and Thr854 are mutated to residues that are more common in the CDD. The most common activating (driver) missense mutations, G719A/C/S and L858R, are not present in the MSA, and the resulting variant is estimated to be much less common than the wt in the conserved TK domain (Table S1). In fact, in only two activating mutations the mutant is more common in the conserved domain than the wt, and in both cases (L861Q and G863D) the position-specific score is 0, indicating that the wt residue is not conserved. This is in accordance with the point of view that these mutations lead to gain-of-function.

### Anaplastic lymphoma kinase

According to our analysis, five out of the six crizotinib-resistant mutants and all 11 neuroblastoma-associated ALK missense mutations lead to a residue that can be observed in related proteins at the same position (in marked difference to driver mutations in EGFR). All of the neuroblastoma-associated mutations involve a change from a residue which is highly conserved in the CDD to one that is uncommon (Table S2), which is also the case for three of the six resistance mutations. Apparently, both resistance and activating mutations in ALK are subject to evolutionary constraints that reduce the mutational landscape.

### Bcr-Abl

#### Single mutations

I have analysed 43 Abl1 mutations carried by CML patients where drug resistance was evident *in vitro*. Remarkably, none of the 43 SNVs is novel, i.e., variations of the same type are evident in related proteins (Table 1 and Table S3), and in all but two cases the change results in a residue that is less conserved in the CDD (in L387F and L387M the mutant has a similar conservation score), which may indicate selective pressure.

#### Compound mutations

**Figure 1:**
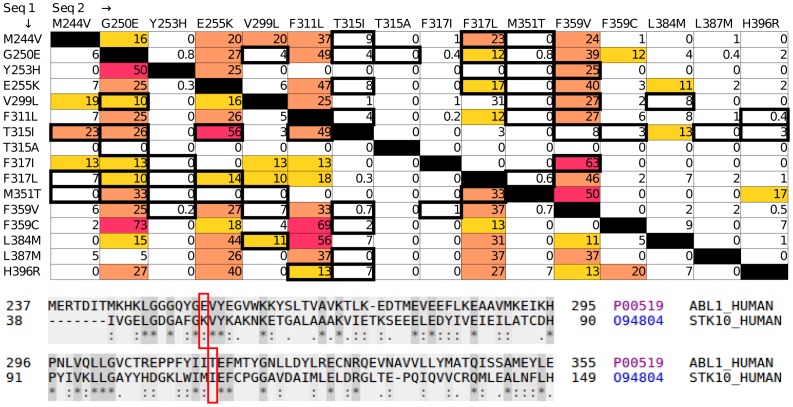
Variations in the evolution of Bcr-Abl1 compound mutations. (Top) Compound mutations are double mutants that arise in the same clone and are detected in treated patients. Using MSA of the Abl1 protein, related sequences where one of the identified mutations is observed as a variation were identified. Each sequence was then analysed in order to examine whether any of the other variations is observed together with the first variation. The results of this analysis are given here as percentage. For example, 50% of the sequences where a His residue is located at a position that is the same as Tyr253 of Abl1 (corresponding to the Y253H mutation) the residue which corresponds to position 250 is Glu (similar to the G250E mutant). Note that the matrix is not symmetric. Taking the same example, only 0.8% of the sequences where Glu is located in the position corresponding to Gly250 in Abl1 (G250E) also posses His in the position corresponding to Tyr253. This difference arises from the relative rarity of the Y250H mutation (0.3%, Table S3) and the relative abundance of the G250E mutation (21%). Compound mutations identified by Khorashad and co-workers [30] are shown within a bold frame. Only double mutants where both single mutations are known to confer drug resistance are analysed, and only residues that are involved in compound mutations reported by Khorashad *et al.* are displayed here; for a full list, see data sheet S8. 102 of 240 possible mutations are not observed in the MSA. The matrix cells are coloured according to the abundance of the conditional variation: less than 10%, white; 10–19%, yellow; 19–50%, orange; more than 50%, red. (Bottom) Sequence alignment between human Abl1 and human STK10. Part of the pairwise alignment between human Abl1 and human STK10 with the location of Abl1 residues Glu255 and Thr315 indicated (red rectangles). The alignment to human STK10 is given as an example, to clarify the findings displayed above. The two residues align with lysine and isoleucine, respectively, corresponding to the E255K/T315I compound mutation. 56% of the sequences that, according to the MSA, have isoleucine at the position corresponding to residue 315 of Abl1, also have lysine at the position corresponding to residue 255.

Recently, Khorashad and co-workers identified a set of double mutations in CML patients treated with TKI [30]. About 70% of those mutations were compound mutations, where the two mutants arise in the same clone of cancer cells. Some of these compound mutations presumably contribute to increased drug-resistance. It is interesting to examine the compound mutations from an evolutionary point of view. Examination of the 21 reported compound mutations [30], reveals that five are completely novel, i.e., a similar (double) variation can not be observed in any of the 1282 sequences homologous to Abl1 (Figure 1 and Table S4). Some of the other 16 variations are quite common. For example, the multiple drug resistance mutant T315I was observed in the same clone with M244V, G250E, E255K, F311L, F359V, F359C, L387M or H396R. 56% of the sequences that, according to the MSA, have isoleucine at the position corresponding to residue 315 of Abl1, also have lysine at the position corresponding to residue 255 - i.e., they align with the T315I/E255K compound mutation (Figure 1, bottom). Note that the order of the occurrence of the mutations may be important, as only 8% of the sequences that correspond to the E255K carry isoleucine at the position corresponding to T315 in Abl1 (compared with 56% if T315I is considered first). Interestingly, when examining all of the possible combinations of the 43 resistant mutants (see data sheet S8) we observe seven variations that are always observed together in natural sequences: (K247N/F317L, E292V/F311I, E292V/F359I, Y253F/T315A, Y253F/F317I, T351A/V379I and Y253F/H375P). These mutations were not reported hitherto, but this may be due to the lack of sensitivity in the sequencing and the small number of patients that were screened. Better sequencing methods [31] are likely to reveal additional compound mutations in Abl1 and other cancer drug targets.

## Discussion

### Most of the resistance mutations are not novel

Analysis of the SNVs leading to drug resistance in EGFR, ALK and Abl1 reveals that in the vast majority of these non-synonymous SNVs (52 of 55, Table 1), a certain residue is modified to one that can be observed in homologous sequences. This may indicate that resistance mutations are subject to purifying selection to some extent. Otherwise, one would expect that novel mutations will be more prevalent.

### Activating mutations tend to favour a change to a less-conserved residue

When it comes to activating mutations, there is a marked difference between the proteins. In EGFR, most of the mutations are novel, which is in line with them being gain-of-function mutations. In ALK, the mutations are not novel. In both cases, however, analysis of the conserved domains reveals that the new variant is almost always less conserved within the domain (Tables S1 and S2). This is in line with the hypothesis that such mutations involve gain-of-function.

### Variability of the mutated residues

In the three proteins surveyed here, some positions have no evolutionary limitations for SNVs, whereas other positions are restricted. For example, six non-synonymous SNVs are possible at the protein level for Ser768 of EGFR. All of these are observed in sequences homologous to EGFR at position 768 (where mutation from Ser to Ile confers drug resistance). On the other hand, threonine in position 790 of the same sequence can only be mutated to methionine or alanine, and the latter is only observed in one sequence. It may be concluded that in the first case, the finding that the resistance mutation is already observed in the evolution is merely a coincidence: after all, all SNVs are possible; whereas in the second case it is meaningful from an evolutionary point of view. An alternative explanation is that all variations in position 768 are possible because they do not lead to a significant reduction in the biological activity of the protein. This reasoning is plausible based on evolutionary theories [32], [33]. To this end, the proportion of *all* non-synonymous SNVs that occur in the three sequences should be considered, and can be compared with the proportion of resistance mutations in which non-synonymous SNVs are observed in the MSA. If non-synonymous SNVs that lead to resistant mutations are subject only to the constraint that they lead to drug resistance and are otherwise evolutionary neutral, one would expect that the corresponding SNVs fall outside of the MSA which describes evolutionary related proteins. If, on the other hand, these SNVs are subject to evolutionary constraints, the vast majority of such SNVs should correspond to residues that can also be identified in other proteins. As shown in Table 2, in the absence of any evolutionary constraints, 1508 non-synonymous SNVs *could be* observed for the kinase domain of EGFR. The 1038 SNVs that *are* observed are 31% less than those possible. Only 5% of the resistance mutations involve SNVs that are not observed in the kinase domain - much less than 31% as would be expected for random SNVs. This is a strong indication that resistance mutations are subject to evolutionary constraints. The expectation value to get the same number of observed resistance mutations at random (i.e., that 52 of the 55 resistance mutations are observed in the MSA due to chance alone, assuming that all non-synonymous SNVs are equally probable) is 5.6E-05.

**Table 2:** The number of possible and observed non-synonymous SNV.

Protein	Kinase domain		% of Resistance mutations
	Possible	Observed	observed in the MSA
EGFR (Total)	1508	1038 (69%)	67%
exons 20–21	679	438 (65%)	100%
ALK	1627	1090 (67%)	83%
Abl1	1541	1254 (81%)	100%
All	4676	3382 (72%)	95%

The total number of possible non-synonymous SNVs, the number of which are observed in the MSA, and the proportion of resistance mutations that are observed in the MSA are shown. For example, if no evolutionary constraints whatsoever had been in effect, 679 non-synonymous SNVs would have been possible in exons 20 and 21 of EGFR. In reality, only 438 are observed. The other 241 non-synonymous SNVs presumably interfere with the biological activity of the enzyme and are selected against. When examining only the residues that are linked to resistance mutations (Table S1), none of the variations falls outside of the MSA. Overall, only 5% of the non-synonymous SNVs that lead to resistance mutations fall within 38% of the SNVs that are possible but not observed in the MSA, which indicates that the resistance mutations are subject to evolutionary constraints.

### Conservation of the mutated residues at the protein level

**Figure 2:**
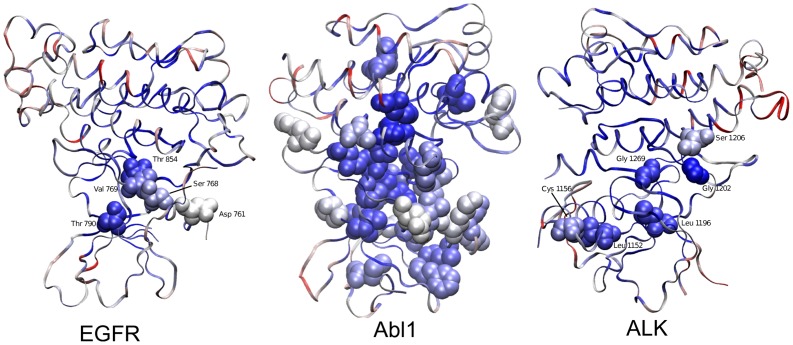
Conservation of resistance mutations at the residue level. The structures of EGFR [68] ALK [69], and Abl1 [70] are shown in a ribbon representation, coloured according to the evolutionary conservation at the residue level. Colouring is at the BWR scale, i.e., highly conserved residues are shown in dark blue, moderately conserved in light blue, mildly conserved or mildly variable in white, moderately variable in pink and highly variable in red. Residues where mutations lead to drug resistance are represented by spheres and indicated (only for EGFR and ALK, note that EGFR residue Leu747 was not resolved in the X-ray structure and is not displayed).

Given that resistance mutations, unlike driver mutations, should not interfere with the biological activity of the protein, one may assume that evolutionary conserved residues will have a lower tendency to be affected (note that residue conservation at the protein level is different than its probability to be observed in the CDD and is not only a function of the number of possible alternations [32]). However, analysis of evolutionary conservation on the protein residue level [34]–[36] reveals that the mutated residues are relatively conserved (Figure 2, Tables S5, S6, S7). This can be explained by reasoning that these residues are either located at the substrate binding site, affect its structure or modify the protein's conformational dynamics; otherwise, mutations cannot lead to drug resistance. Wide differences between the individual residues are observed, however. Some residues are highly conserved (e.g., the gatekeeper residues Thr854 in EGFR, Leu1196 in ALK and Thr315 in Abl1), whereas others are somewhat variable. Interestingly, the median variability score is higher for the activating mutations in EGFR and ALK than for the resistance mutations, indicating that a mutation of a conserved residue is more likely to yield a drug-resistance mutant. This finding is somewhat counter-intuitive because driver mutations are expected to yield functions that are important for tumour growth or proliferation [37], and it is therefore reasonable to expect that they would tend to occur at conserved sites and will not be so sensitive to evolutionary constraints.

Given that, by and large, resistance mutations occur at conserved sites, how is the protein able to maintain its function? A possible explanation is that the resistance mutations are conservative, i.e., they involve modifications of amino acid residues that do not alter their biochemical properties. Empirical quantification of the biochemical distance between residues was suggested by Grantham [38], who devised a metrics known as the ‘Grantham distance’. The more radical the substitution, the higher is the Grantham distance. Thus, the Grantham distance between isoleucine and leucine is 5, whereas cysteine and phenylalanine are 205 Grantham units away from each other. The mean Grantham distance is 100, corresponding to the biochemical difference between phenylalanine and histidine, indicating that most of the possible alternations are radical rather than conservative. Examination of the Grantham distances (Table S5, Table S6 and Table S7) reveals that both radical and conservative mutations are observed. The median Grantham distance, however, is higher for resistance mutations than for activating mutations in EGFR and ALK. Interestingly, the median Grantham distance for resistance mutations in Abl1 is quite small (51). This indicates that relatively small changes at the binding site already lead to drug resistance, and explains why drug resistance due to point mutations is so common in CML.

### Resistance mutations are subject to evolutionary constraints

Bioinformatic analysis of resistance mutations in EGFR, ALK and Abl1 reveals that although many non-synonymous SNVs are possible, few of the drug resistance mutations are novel in the sense that similar variations were not observed in the evolution. This limits the potential number of SNVs by 19–35%, depending on the protein (Table 2). Further limitation comes from the fact that resistance mutations are more likely to occur at conserved residues, although they can also involve mildly conserved-residues. Comparison between resistant and activating mutations in EGFR and ALK indicates that the resistant mutations are more likely to be radical from a biochemical point of view. This may have implications for example when whole genomes of treated cancer patients are analysed for unknown mutations and there is a need to separate between driver mutations, passenger mutations and resistance mutations.

### Compound mutations

Compound mutations that lead to drug resistance typically involve a combination of two single resistance mutations that together lead to improved drug resistance and may result in relapse over treatment. The analysis of compound mutations lead to two conclusions. First, 24% of the known compound mutations are not observed *together* in any Abl1 homologue, whereas all single mutations were observed. This may indicate that multiple mutations that do not significantly impair the function of the enzyme are not subject to additional evolutionary pressure that would prevent their accumulation. Alternatively, the accumulation of multiple mutations may be slightly deleterious which does not prevent them from being fixed [39]. Second, several alternations seem to occur together in homologous sequences but have hitherto not been identified in patients, either due to experimental limitations, small sample sizes, or because they are less beneficial for resistance. Both findings suggest that additional compound resistance mutations will be reported in the future, in Abl1 and other genes, and will be difficult to target. Moreover, compound mutations have recently been observed also in the context of EGFR activating mutations [40], [41], further indicating that such mutations should be expected in other genes.

Our understanding of cancer evolution is becoming better owing to better sequencing methods [42], new analysis tools for cancer gene networks [43] and development of evolutionary models [44]–[47]. Several methods are available for distinguishing between driver and passenger mutations [37], [48]–[51]. Many studies demonstrate the necessity for taking the evolutionary forces that drive cancer progression into account [52]–[58]. In this article it is shown that evolutionary reasoning should also be considered for the analysis of resistance mutations.

## Methods

### Analysis of sequence variations in EGFR, ALK and Abl1

Analysis of the sequence variations in the three drug targets EGFR, ALK and Abl1 was performed as follows. First, the protein sequences (accession numbers: EGFR, NP_005219.2; ALK, AAB71619.1 and Abl1, NP_005148.2) were downloaded from www.ncbi.nlm.nih.gov. The tyrosin kinase (TK) domains of each protein were extracted. These domains correspond to EGFR residues 713–968, ALK residues 1109–1385, and Abl1 residues 235–497. Homologous sequences were identified by using the recently developed DELTA-BLAST method [59], employing a threshold of 500 sequences for EGFR and ALK and 2000 for Abl1 (using additional sequences for EGFR and ALK did not significantly modify the results). Homologous sequences were identified in the Swiss-Prot database of manually curated proteins [60], www.uniprot.org. A representative set of similar sequences for each protein was prepared by removing nearly identical sequences based on a 95% similarity criterion, using the skipredundant program available from EMBOSS [61], http://emboss.sourceforge.net, which employs the global alignment algorithm of Needleman and Wunsch [62]. The remaining sequences (276 for EGFR, 273 for ALK, and 1282 for Abl1) were aligned together by use of the FFT-NS-2 method within the MAFFT program [63]. The GUIDANCE program [64] was used to provide an estimated accuracy for each position in the multiple sequence alignment (MSA), and generate a more robust MSA. MAFFT and the FFT-NS-2 algorithm were used within GUIDANCE. The size of the ALK MSA was too large to employ GUIDANCE on this set of sequences.

### Evolutionary conservation at the residue level

The Consurf server [35], [36] was used to estimate the evolutionary conservation at the residue level for EGFR, ALK and Abl1. Multiple sequence alignment within Consurf was built with MAFFT [63]. For each protein, up to 500 homologues were collected from Swiss-Prot [60]. The CS-Blast algorithm [65], [66] was employed to search for homologues. Default parameters were used otherwise. Protein figures were generated with VMD [67].

## Supporting Information

Table S1 **Analysis of drug-resistant and drug-sensitive mutants of EGFR.** The most common mutations [18] are shown in bold-face. *^a^* Confidence scores are between 0 and 1, where 1 means robust, see [64]. Guidance scores are given for the position. *^b^* PSSM = position specific scoring matrix. Log-odds scores calculated as the log (base 2) of the observed substitution frequency at a given position divided by the expected substitution frequency at that position. Positive scores for a given residue indicate that it is more common at a given site than expected for a random protein sequence. *^c^* NP = not present. The CDD domain is CDD:173654. Tyrosine kinase, catalytic domain.
(PDF)

Table S2 **Analysis of ALK drug-resistant mutations (lung cancer) and activating mutations (neuroblastoma).** Only mutations in the catalytic domain are analysed. See the legend of Table S1 for explanation on the scores. The CDD domain is cd05036, catalytic domain of the Protein Tyrosine Kinases, Anaplastic Lymphoma Kinase and Leukocyte Tyrosine Kinase. The most medically relevant mutations are shown in bold face. NP = not present.
(PDF)

Table S3 **Analysis of Abl-1 drug-resistant mutations.** Only mutations in the catalytic domain are analysed. See the legend of Table S1 for explanation on the scores. The CDD domain is cd05052, catalytic domain of the protein tyrosine kinase.
(PDF)

Table S4 **Analysis of Abl-1 drug-resistant compound mutations.** The frequency of sequences in the MSA that carry out the indicated double mutants that have shown to occur in the same clone in patients [30] is shown. See also Figure 1 of the main text.
(PDF)

Table S5 **Evolutionary analysis of drug-resistant and drug-sensitive mutants of EGFR.** Grantham distances [38] and Consurf conservation scores [34], [36] are shown for each mutation. Mutations that are observed in the MSA are underlined. Lower (negative) values indicate conserved residues. The average Grantham distance between pairs of amino acids, if one takes into account all possible substitutions, is 100. Median Consurf score are calculated per residues, i.e., if several non-synonymous SNVs are observed for a residue it is only counted once. Variations that are observed in the MSA (see Table S1) are underlined.
(PDF)

Table S6 **Evolutionary analysis of drug-resistant and drug-sensitive mutants of ALK.** Grantham distances [38] and Consurf conservation scores [34], [36] are shown for each mutation.
(PDF)

Table S7 **Evolutionary analysis of drug-resistant and drug-sensitive mutants of Abl11.** Grantham distances [38] and Consurf conservation scores [34], [36] are shown for each mutation.
(PDF)

Data S1 **Variations in the evolution of all Bcr-Abl1 compound mutations.** This tab-delimited file can be read by text editor and spreadsheet programs such as LibreOffice Calc or Microsoft Excel, and provides the same information as given in figure 1 for *all* of the possible combination of the 43 resistant mutants.
(CSV)

Data S2 **T**his data file contains a list of the sequences that were aligned to EGFR, ALK and Abl1.
(ZIP)
