# Supplementary material for: Drug Resistance Missense Mutations in Cancer Are Subject to Evolutionary Constraints
Source: PLoS One. 2013 Dec 20;8(12):e82059. doi: 10.1371/journal.pone.0082059 (PMC3869674; doi:10.1371/journal.pone.0082059)
Supplement: Table S1 — Analysis of drug-resistant and drug-sensitive mutants of EGFR. The most common mutations [18] are shown in bold-face. a Confidence scores are between 0 and 1, where 1 means robust, see [64]. Guidance scores are given for the position. b PSSM = position specific scoring matrix. Log-odds scores calculated as the log (base 2) of the observed substitution frequency at a given position divided by the expected substitution frequency at that position. Positive scores for a given residue indicate that it is more common at a given site than expected for a random protein sequence. c NP = not present. The CDD domain is CDD:173654. Tyrosine kinase, catalytic domain. (PDF) [file pone.0082059.s001.pdf]

Table S1

**Mutation**	**Prevalence in the MSA (%)**	**Guidance score**[a]	**Wt CDD PSSM score**[b]	**Mutant PSSM score**	**Number of possible mutations**	**Number of observed mutations**
Resistance mutations:						
Exon 19:						
L747S	NP[c]	0.96	5	-1	4	3
D761Y	NP	0.95	1	-6	7	5
Exon 20:						
S768I	1.4	0.99	1	-7	6	6
V769L	44	0.94	0	5	5	3
**T790M**	31	0.99	3	8	6	2
Exon 21:						
T854A	53	1.00	2	4	6	3
Activating mutations:						
Exon 18:						
**G719A**	0.7	1	7	-2	6	2
**G719C**	NP	1	7	-6	6	2
**G719S**	NP	1	7	-3	6	2
S720P	NP	1	2	-6	6	5
Exon 20:						
V765A	2	0.99	8	-5	5	3
T783A	4	0.70	0	-2	5	5
Exon 21:						
N826S	7	1	2	0	7	7
A839T	NP	1	7	-5	6	1
K846R	1	0.95	1	-5	7	6
**L858R**	NP	0.99	6	-7	5	2
L861Q	1	0.85	0	0	5	2
G863D	3	0.65	0	1	6	3
