# Supplementary material for: Drug Resistance Missense Mutations in Cancer Are Subject to Evolutionary Constraints
Source: PLoS One. 2013 Dec 20;8(12):e82059. doi: 10.1371/journal.pone.0082059 (PMC3869674; doi:10.1371/journal.pone.0082059)
Supplement: Table S2 — Analysis of ALK drug-resistant mutations (lung cancer) and activating mutations (neuroblastoma). Only mutations in the catalytic domain are analysed. See the legend of Table S1 for explanation on the scores. The CDD domain is cd05036, catalytic domain of the Protein Tyrosine Kinases, Anaplastic Lymphoma Kinase and Leukocyte Tyrosine Kinase. The most medically relevant mutations are shown in bold face. NP = not present. (PDF) [file pone.0082059.s002.pdf]

Table S2

**Mutation**	**Prevalence in the MSA (%)**	**Guidance score**^{a}	**Wt CDD PSSM score**^{b}	**Mutant PSSM score**	**Number of possible mutations**	**Number of observed mutations**
TKI resistant mutations:						
L1152R	NP	0.97	4	2	5	3
C1156Y	7	0.30	8	-3	7	6
L1196M	32	1	5	1	5	3
G1202R	0.4	0.97	7	-3	5	2
S1206Y	0.4	0.98	4	-4	6	3
G1269A	48	1	4	4	5	2
Neuroblastoma mutations:						
T1151M	15	0.98	5	-2	6	5
I1171N	0.4	1	4	-3	7	4
F1174C	8	0.92	8	-3	7	7
F1174I	11	0.92	8	-1	7	7
**F1174L**	37	0.92	8	0	7	7
F1174V	7	0.92	8	-2	7	7
F1245C	11	0.99	7	-3	6	6
F1245L	6	0.99	7	-1	6	6
F1245V	16	0.99	7	-2	6	6
**R1275Q**	0.4	0.92	6	0	5	3
Y1278S	5	0.53	8	-3	7	6
