# Supplementary material for: Drug Resistance Missense Mutations in Cancer Are Subject to Evolutionary Constraints
Source: PLoS One. 2013 Dec 20;8(12):e82059. doi: 10.1371/journal.pone.0082059 (PMC3869674; doi:10.1371/journal.pone.0082059)
Supplement: Table S3 — Analysis of Abl-1 drug-resistant mutations. Only mutations in the catalytic domain are analysed. See the legend of Table S1 for explanation on the scores. The CDD domain is cd05052, catalytic domain of the protein tyrosine kinase. (PDF) [file pone.0082059.s003.pdf]

Table S3

**Mutation**	**Prevalence in the MSA (%)**	**Wt CDD PSSM score**[b]	**Mutant PSSM score**	**Number of possible mutations**	**Number of observed mutations**
M244V	13	8	0	6	6
K247N	1	6	-1	7	7
L248V	10	5	0	5	2
G250E	21	6	-3	5	5
Q252H	2	7	0	7	7
Y253H	0.3	8	1	7	6
Y253F	64	8	3	7	6
E255K	23	6	0	7	7
E255V	15	6	-3	7	7
L273F	3	5	0	6	5
E279K	4	3	0	7	7
E282K	5	6	0	7	7
K285N	9	4	-1	7	7
V289L	24	4	1	6	4
E292K	20	5	0	7	7
E292V	0.2	5	-3	7	7
N297T	0.2	7	-1	6	5
V299L	8	5	0	5	3
F311I	23	7	-1	6	6
F311L	41	7	0	6	6
T315A	0.1	6	-1	5	4
**T315I**	3	6	-1	5	4
F317C	0.9	7	-3	6	6
F317I	0.6	7	-1	6	6
F317L	26	7	0	6	6
F317S	0.3	7	-3	6	6
F317V	0.3	7	-1	6	6
S349L	10	5	-2	5	4
M351T	0.5	8	-1	6	4
F359C	3.5	7	-3	6	6
F359I	39	7	-1	6	6
F359V	33	7	-1	6	6
H375P	6	7	-2	7	6
V379I	47	5	3	5	3
L384M	6	5	0	5	4
L387F	10	4	4	6	6
L387M	3	4	4	6	6
H396R	1	8	-1	7	7
H396P	3	8	3	7	7
T406I	11	6	-1	5	5
W430L	2	12	-2	6	6
F486S	3	7	-3	6	6
