# Supplementary material for: Drug Resistance Missense Mutations in Cancer Are Subject to Evolutionary Constraints
Source: PLoS One. 2013 Dec 20;8(12):e82059. doi: 10.1371/journal.pone.0082059 (PMC3869674; doi:10.1371/journal.pone.0082059)
Supplement: Table S4 — Analysis of Abl-1 drug-resistant compound mutations. The frequency of sequences in the MSA that carry out the indicated double mutants that have shown to occur in the same clone in patients [30] is shown. See also Figure 1 of the main text. (PDF) [file pone.0082059.s004.pdf]

Table S4

Mutation	Frequency in seqs with the first variation (%)	Frequency in seqs with the second variation (%)
M244V/T315I	9	23
M244V/F317L	23	7
M244V/M351T	0	0
G250E/V299L	4	10
G250E/M351T	0.8	33
G250E/T315I	4	23
G250E/T315A	0	0
G250E/F317L	12	10
Y253H/F317L	0	0
Y253H/F359V	25	0.2
E255K/T315I	8	56
E255K/F317L	17	14
V299L/M351T	0	0
V299L/F359V	27	7
V299L/L384M	8	11
F311L/T315I	4	49
F311L/H396R	0.4	13
T315I/F359C	3	2
T315I/L387M	0	0
T315I/H396R	3	7
F317L/M351T	0.6	33
