# Supplementary material for: Drug Resistance Missense Mutations in Cancer Are Subject to Evolutionary Constraints
Source: PLoS One. 2013 Dec 20;8(12):e82059. doi: 10.1371/journal.pone.0082059 (PMC3869674; doi:10.1371/journal.pone.0082059)
Supplement: Table S5 — Evolutionary analysis of drug-resistant and drug-sensitive mutants of EGFR. Grantham distances [38] and Consurf conservation scores [34], [36] are shown for each mutation. Mutations that are observed in the MSA are underlined. Lower (negative) values indicate conserved residues. The average Grantham distance between pairs of amino acids, if one takes into account all possible substitutions, is 100. Median Consurf score are calculated per residues, i.e., if several non-synonymous SNVs are observed for a residue it is only counted once. Variations that are observed in the MSA (see Table S1) are underlined. (PDF) [file pone.0082059.s005.pdf]

Table S5

**Mutation**	**Grantham distance**	**Consurf normalised score**
Resistant mutations:		
Exon 19:		
L747S	145	-0.503
D761Y	160	0.930
Exon 20:		
S768I	142	-0.049
V769L	32	-0.452
T790M	81	-0.967
Exon 21:		
T854A	58	-0.986
Median	112	-0.478
Activating mutations:		
Exon 18:		
G719A	60	-1.346
G719C	159	
G719S	56	
S720P	74	0.799
Exon 20:		
V765A	64	-0.091
T783A	58	1.448
Exon 21:		
N826S	46	1.194
A839T	58	-1.339
K846R	26	-0.229
L858R	102	-0.885
L861Q	113	0.368
G863D	94	1.675
Median	62	0.139
