# Supplementary material for: Drug Resistance Missense Mutations in Cancer Are Subject to Evolutionary Constraints
Source: PLoS One. 2013 Dec 20;8(12):e82059. doi: 10.1371/journal.pone.0082059 (PMC3869674; doi:10.1371/journal.pone.0082059)
Supplement: Table S6 — Evolutionary analysis of drug-resistant and drug-sensitive mutants of ALK. Grantham distances [38] and Consurf conservation scores [34], [36] are shown for each mutation. (PDF) [file pone.0082059.s006.pdf]

Table S6

Mutation	Grantham distance	Consurf normalised score
TKI resistant mutations:		
L1152R	102	-0.791
C1156Y	112	-0.272
L1196M	15	-0.857
G1202R	125	-1.306
S1206Y	177	0.020
G1269A	60	-0.949
Median	107	-0.824
Neuroblastoma mutations:		
T1151M	81	-0.690
I1171N	149	-0.987
F1174C	205	-0.462
F1174I	21	
F1174L	22	
F1174V	50	
F1245C	205	-0.300
F1245L	22	
F1245V	50	
R1275Q	43	-1.150
Y1278S	144	-0.143
Median	50	-0.576
