# Supplementary material for: Drug Resistance Missense Mutations in Cancer Are Subject to Evolutionary Constraints
Source: PLoS One. 2013 Dec 20;8(12):e82059. doi: 10.1371/journal.pone.0082059 (PMC3869674; doi:10.1371/journal.pone.0082059)
Supplement: Table S7 — Evolutionary analysis of drug-resistant and drug-sensitive mutants of Abl11. Grantham distances [38] and Consurf conservation scores [34], [36] are shown for each mutation. (PDF) [file pone.0082059.s007.pdf]

Table S7

Mutation	Grantham distance	Consurf normalised score
M244V	21	-0.149
K247N	94	0.410
L248V	32	-0.639
G250E	98	-0.136
Q252H	24	-0.342
Y253H	83	-1.070
Y253F	22	
E255K	56	-0.302
E255V	121	
L273F	22	-0.530
E279K	56	0.155
E282K	56	-0.146
K285N	94	0.514
V289L	32	0.026
E292K	56	0.956
E292V	121	
N297T	65	-0.702
V299L	32	-0.589
F311I	21	-0.216
F311L	22	
T315A	58	-0.740
T315I	89	
F317C	205	0.481
F317I	21	
F317L	22	
F317S	155	
F317V	50	
S349L	145	0.131
M351T	81	-1.100
F359C	205	-0.665
F359I	21	
F359V	22	
H375P	100	0.624
V379I	29	-0.815
L384M	15	-0.847
L387F	22	-0.040
L387M	15	
H396R	29	0.516
H396P	100	
T406I	89	-0.935
W430L	61	-0.494
F486S	155	-0.979
Median	56	-0.302
